# Long-term serological follow-up after primary *Coxiella burnetii* infection in patients with vascular risk factors for chronic Q fever

**DOI:** 10.1007/s10096-021-04179-5

**Published:** 2021-02-10

**Authors:** Sheila B. Buijs, Sanne K. Stuart, Jan Jelrik Oosterheert, Steffi Karhof, Andy I. M. Hoepelman, Nicole H. M. Renders, André S. van Petersen, Chantal P. Bleeker-Rovers, Peter C. Wever, Olivier H. J. Koning

**Affiliations:** 1 Department of Internal Medicine and Infectious Diseases, University Medical Center Utrecht, Utrecht University, Utrecht, The Netherlands; 2 Department of Surgery, Jeroen Bosch Hospital, ’s-Hertogenbosch, The Netherlands; 3 Department of Medical Microbiology and Infection Control, Jeroen Bosch Hospital, ’s-Hertogenbosch, The Netherlands; 4 Department of Surgery, Bernhoven Hospital, Uden, The Netherlands; 5 Department of Internal Medicine and Infectious Diseases, Radboud Expert Centre for Q Fever, Radboud University Medical Center, Nijmegen, The Netherlands

**Keywords:** *Coxiella burnetii*, Q fever, Serology, Aneurysm, Blood vessel prosthesis

## Abstract

We evaluated the long-term serological follow-up of patients with vascular risk factors for chronic Q fever that were previously *Coxiella* *burnetii* seropositive. *C. burnetii* phase I IgG titers were reevaluated in patients that gave informed consent or retrospectively collected in patients already deceased or lost to follow-up. Of 107 patients, 25 (23.4%) became seronegative, 77 (72.0%) retained a profile of past resolved Q fever infection, and five (4.7%) developed chronic Q fever. We urge clinicians to stay vigilant for chronic Q fever beyond two years after primary infection and perform serological testing based on clinical presentation.

## Introduction

Q fever is a zoonosis caused by *Coxiella burnetii*. Persistent or chronic Q fever infection develops in 1–5% of patients after primary infection. Risk factors for chronic Q fever are the presence of an aneurysm, vascular prosthesis, cardiac valvulopathy, or valvular surgery [1, 2]. Therefore, the Centers for Disease Control and Prevention (CDC) advises serological follow-up for two years in these high-risk patients after acute Q fever infection [3].

From 2007 to 2010, the largest Q fever outbreak ever reported occurred in the Netherlands. Following the outbreak, a growing number of patients have been diagnosed with chronic Q fever. Currently, 519 chronic Q fever patients have been identified with a vascular focus of infection in 47.0% [4]. In 69% of Dutch patients, a chronic Q fever-related complication was already present at diagnosis [4]. To detect and treat chronic Q fever earlier, screening programs in patients with risk factors were initiated after the Dutch epidemic. One program focused on patients with an aneurysm and/or vascular prosthesis: during the study period from 2009 to 2013, 40 (30.8%) of 130 *C. burnetii* seropositive patients were diagnosed with chronic Q fever [5]. An additional three seropositive patients were diagnosed during serological follow-up thereafter, but the follow-up time was shorter than the two years the CDC recommends [6].

Thus, the long-term risk for chronic Q fever is still unknown in high-risk Dutch patients with vascular disease. Therefore, we performed long-term serological follow-up of patients with vascular risk factors for chronic Q fever that were found to be *C. burnetii* seropositive during screening in 2009–2013.

## Patients and Methods

### Study population

Patients with abdominal aortic and/or iliac disease at the outpatient clinics of the Jeroen Bosch Hospital and Bernhoven Hospital that were screened for chronic Q fever between 2009 and 2013 and had serological evidence of *C. burnetii* infection were included in this study. Aorto–iliac disease was defined as an abdominal aortic aneurysm (≥30 mm), an aneurysm of the common iliac artery (>12 mm), or an aorto–iliac reconstruction. These patients were approached by their treating physician between December 2018 and May 2019. Written information was given and informed consent was obtained. Data on patient and disease characteristics were collected from the electronic medical records. Data were stored anonymously in an SPSS database, version 25.0.0.2.

### Microbiological testing

Phase I and phase II IgG *C. burnetii* antibodies were measured on serum samples using indirect immunofluorescence assay (IFA) (Focus Diagnostics, Cypress, CA, USA). PCR was performed for detection of *C. burnetii* DNA in serum or tissue samples (in-house, real-time PCR targeting the repetitive element IS1111) [7]. Based on serology results, patients could be classified into three groups: (1) seronegative for Q fever, (2) past resolved Q fever, or (3) chronic Q fever. Chronic Q fever is further classified into proven, probable, or possible chronic Q fever according to the definitions formulated by the Dutch Q Fever Consensus Group [8].

### Statistical analysis

Descriptive data were generated and analyzed using SPSS version 25.0.0.2. Continuous variables were compared using the independent sample *t* test or Kruskal–Wallis test as appropriate. Categorical data were compared using Fisher’s exact test or chi-square test as appropriate. A *p*-value of <.05 was considered statistically significant.

### Ethical statement

This study was performed in line with the principles of the Declaration of Helsinki. Approval was granted by the Medical Ethics Committee Brabant (NL67239.028.18). Written informed consent for serological follow-up was obtained from all participants.

## Results

A total of 148 patients with aorto–iliac disease previously reported to be *C. burnetii* seropositive were identified. During this study, 18 patients (12.2%) declined participation. Of the remaining 130 patients, 48 (36.9%) gave informed consent for serological reevaluation, 20 patients (15.4%) were lost to follow-up, and 62 (47.7%) had died in the time between initial screening and this study. Routine care serological results were available for 17/20 patients lost to follow-up and 42/62 deceased patients. Thus, serological follow-up was available in 107 patients at any given time after initial screening.

### Serological follow-up

Most patients were male (86, 80.4%), and 103 (96.3%) had past resolved Q fever infection at the start of screening (Table 1). After a median follow-up of 64 months (IQR, 28.0–80.5), 25 patients (23.4%) had become seronegative for *C. burnetii*, but in most patients (72.0%), a serological profile of past resolved Q fever infection remained (Table 1). Five patients (4.7%) developed chronic Q fever. Of these, four developed chronic Q fever within one year after initial screening and were identified during routine follow-up. One patient was diagnosed during the current long-term serological follow-up study, 63 months after having an initial low phase I IgG titer of 1 : 64 in 2013.

**Table 1.** Patient characteristics of all patients: reevaluated, lost to follow-up, and deceased patients

	All patients (*n*=107)	Reevaluated (*n*=48)	Lost to FU (*n*=17)	Deceased (*n*=42)
Male gender (%)	86 (80.4)	39 (81.3)	12 (70.6)	35 (83.3)
Mean age at start screening, years (sd)	70.2 (8.5)	66.5 (8.5)	71.1 (7.7)	74.1 (7.2)
Diagnosis at start screening*				
- Acute Q fever (%)	2 (1.9)	2 (4.2)	-	-
- Past resolved (%)	103 (96.3)	45 (93.8)	17 (100)	41 (97.6)
- Phase I IgG ≥1.024 (%)	2 (1.9)	1 (2.1)	-	1 (2.4)
Reason of start screening: aneurysm (%)	64 (59.8)	31 (64.6)	12 (70.6)	21 (50.0)
- Asymptomatic (%)	58 (54.2)	30 (62.5)	10 (58.8)	18 (42.9)
- Symptomatic (%)	1 (0.9)	-	1 (5.9)	-
- Rupture (%)	5 (4.7)	1 (2.1)	1 (5.9)	3 (7.1)
Reason of start screening: vascular reconstruction (%)	43 (40.2)	17 (35.4)	5 (29.4)	21 (50.0)
- EVAR (%)	11 (10.3)	4 (8.3)	1 (5.9)	6 (14.3)
- Aorta bifurcation graft (%)	20 (18.7)	9 (18.8)	2 (11.8)	9 (21.4)
- Aortic tube graft (%)	11 (10.3)	4 (8.3)	2 (11.8)	5 (11.9)
- Iliofemoral bypass graft (%)	1 (0.9)	-	-	1 (2.4)
Median FU time, months (IQR)	64.0 (28.0–80.5)	81.0 (70.8–91.3)	43.0 (17.0–64.0)	28.0 (18.0–47.8)
Number of phase I IgG measurements per patient, median (IQR)	5.0 (3.0–7.0)	5.5 (3.8–9.0)	5.0 (3.0–5.0)	4.0 (3.0–6.8)
Diagnosis at end of follow-up*				
- Seronegative (%)	25 (23.4)	10 (20.8)	5 (29.4)	10 (23.8)
- Past resolved (%)	77 (72.0)	35 (72.9)	12 (70.6)	30 (71.4)
- Chronic Q fever (%)	5 (4.7)	3 (6.3)	-	2 (4.8)
Median time to, months (IQR)				
- Seronegativity	21.0 (9.0–36.0)	45.0 (16.5–64.5)	33.0 (21.0–36.0)	9.0 (6.0–15.8)
- Chronic Q fever diagnosis	9.0 (3.0–12.0)	12.0 (7.5–37.5)	NA	6.0 (4.5–7.5)

Abbreviations: *FU*, follow-up; *sd*, standard deviation; *EVAR*, endovascular aneurysm repair

^*^Definition of different serological profiles: negative for Q fever, phase I IgG <1:32 and phase II IgG <1:32; acute Q fever, phase I IgM and/or phase II IgM >1:32, with a combination of phase I IgG, phase II IgG and/or a positive polymerase chain reaction not corresponding with other serological profiles; past resolved Q fever infection, phase II IgG ≥1:32, with phase I IgG <1:1 024; chronic Q fever, phase I IgG ≥1:1 024

## Discussion

Serological follow-up of 107 high-risk patients previously infected with *C. burnetii* revealed that 25 patients (23.4%) became *C. burnetii* seronegative, while the majority (72.0%) remained seropositive with a profile of past resolved Q fever infection, and five (4.7%) developed chronic Q fever. In 48 patients, serology was reevaluated during this study, identifying one (2.1%) new patient with proven chronic Q fever. This patient was diagnosed with chronic Q fever more than five years after having an initial low phase I IgG titer of 1 : 64 at screening in 2013.

In another long-term follow-up study performed in 2016–2017 among 133 patients with cardiac valvulopathy in the Netherlands, six patients (4.5%) were found to have a chronic Q fever infection [9]. In contrast to our study, seroprevalence was only measured during the study period in 2016–2017 without knowledge of serological results before this study period. Theoretically, some of these patients could have been primarily infected in the years after the major Q fever outbreak. Two other long-term population-based follow-up studies were performed in patients with a primary infection during the Dutch Q fever outbreak, regardless of the presence of risk factors for chronic Q fever. In these studies, 0.5% and 0.6% of screened patients developed chronic Q fever at four and seven years follow-up, respectively [10, 11].

We show here that many years after a large Q fever epidemic, chronic Q fever cases can still be detected during serological follow-up. The effect of long-term follow-up is greater when performed in previously infected patients with risk factors for chronic Q fever. Knowing our study population was derived from a cohort of originally 1032 patients with aorto–iliac disease [6], the long-term detection rate among high-risk patients in whom the serological status is unknown is estimated to be about 0.1%. In screening programs performed earlier after the outbreak, the percentage of high-risk patients with unknown serological status that were diagnosed with chronic Q fever was higher (1.6–4.2%) [6, 12]. Currently, a nationwide case finding program is being performed in high-risk patients in the Netherlands after this program was expected to be cost-effective [13].

A strength of our study is the long follow-up period starting as early as 2009. Therefore, *C. burnetii* seropositivity in these patients is in all probability the result of a primary infection during the 2007–2010 Q fever outbreak. To our knowledge, this is the first study to describe a long-term serological follow-up of vascular high-risk patients many years after their primary Q fever infection. The high dropout percentages in our study are probably the result of the long period between this study and the initial screening. Another limitation is the gap in measurements of serological titers between screening and this long-term follow-up study. Therefore, we do not know the precise course of serological titers.

Serological follow-up in the four patients diagnosed with chronic Q fever during routine care was performed after initial screening showed an acute Q fever infection (*n*=1) or a high phase I IgG of 1 : 512 (*n*=3), confirming the recommendation that serological follow-up in these cases is necessary. However, the one patient newly diagnosed with chronic Q fever in this study emphasizes the need to remain alert for chronic Q fever. This patient had no specific symptoms of either an acute or chronic infection and no Q fever-related complications occurred, leaving the precise interval between acute and chronic infection unknown.

We recommend performing serological testing beyond the recommended two years based on clinical information that can range from vague symptoms as fatigue to diagnoses of mycotic aneurysms or culture-negative endocarditis. In case of a new epidemic, the CDC guideline of two-year serological follow-up should be followed in every patient with acute Q fever [3], but we also recommended to screen high-risk patients for asymptomatic primary infection and perform two-year serological follow-up in those who are then found to be *C. burnetii* seropositive.

## Conclusions

In a cohort of patients with aorto–iliac disease previously reported to be *C. burnetii* seropositive, most patients (72.0%) remained seropositive and five (4.7%) developed chronic Q fever: one patient with proven chronic Q fever was diagnosed more than five years after having an initial low phase I IgG of 1 : 64. We urge clinicians to stay vigilant for chronic Q fever and perform serological testing based on clinical presentation.

## Data Availability

The datasets generated and analyzed during the current study are available from the corresponding author on reasonable request.
